# Role of resilience and self-coping strategy in determining positive emotions during pandemic among healthcare professionals in Iran

**DOI:** 10.1186/s40359-023-01323-y

**Published:** 2023-09-22

**Authors:** Remya Lathabhavan, Teena Bharti, Zohreh Hosseini Marznaki

**Affiliations:** 1 OB and HRM Area, Indian Institute of Management, Bodh Gaya, Bihar, India; 2 Department of Nursing, Amol Faculty of Nursing and Midwifery Sciences, Mazandaran University of Medical Sciences, Sari, Iran

**Keywords:** Resilience, Self-coping strategy, Fear of COVID-19, Healthcare professionals, Nurses, Iran

## Abstract

**Background:**

The unprecedented COVID-19 pandemic placed immense stress on healthcare professionals worldwide. This strain often resulted in imbalances in their physical and mental well-being, necessitating effective coping mechanisms. In light of this context, this study investigates the correlations between the fear of COVID-19, self-coping strategies, and positive emotions, with particular reference to the Iranian health care industry.

**Methods:**

Data were collected from 1050 healthcare professionals in Iran and the data were analysed sing structural equation modelling.

**Results:**

The study revealed that pandemic fear negatively impacts self-coping strategies and positive emotions during crisis scenarios. Conversely, self-coping strategies have a positive correlation with positive emotions. The research also underscored the role of resilience in reinforcing the favorable link between self-coping strategies and positive emotions.

**Conclusions:**

This study is one of the first to explore the significance of resilience and self-coping strategies among Iranian healthcare professionals during the pandemic. Its findings offer valuable insights for researchers and practitioners, paving the way for further contributions in this field. Future research endeavors may consider investigating the effects of various psychological interventions, including breathing techniques, self-talk, physical exercises, yoga, optimizing sleep, and dietary measures on the resilience and self-coping practices of healthcare professionals.

## Introduction

The global impact of COVID-19 on underprepared healthcare systems has been unprecedented. Healthcare systems worldwide faced not only the physical demands of managing the physiological and medical aspects of the outbreak but also grappled with the emotional and psychological challenges linked to the disease and its consequences [1]. One of the biggest challenges during the peak of the pandemic was the shortage of healthcare workers, especially in lower-middle and low-income countries; the World Health Organization (WHO) estimated a shortage of 15 million healthcare professionals in these countries in 2020. Accounting for population size, high-income countries had 6.5 times more healthcare workers per 10,000 people than low-income countries [2]. In contrast to the disparities in healthcare professional availability among high, low, and middle-income countries, research indicated that the prevalence of COVID-19 was three times higher in high-income countries compared to other nations, with rates of 17,371 cases per 1 million population versus 6180 cases per 1 million population [3]. According to the Global statistics in November 2022, while there were 630,387,8583 confirmed cases of COVID-19, including 6,583,163 deaths across the globe, the Islamic Republic of Iran reported 7,558,593 confirmed cases of COVID-19 with 144,604 deaths [4]. Despite lower pandemic numbers, the strain imposed on the overworked health workers in countries like Iran cannot be ignored.

Frontline healthcare workers (HCWs), who serve as the first point of contact in health emergencies, face significant exposure to psychological distress, often resulting in fear, anxiety, marginalization, prejudice, and depression [5, 6]. Given the clinical presentation, epidemiological characteristics, rapid transmission pattern, severity of public health impact, novelty, scale, and implications for global public health, the pandemic carried a high potential for a multitude of psychological issues, not only to the general public but to the health workers as well [7]. In addition, HCWs faced additional problems such as job insecurity and increased workloads [8]. The effects of these multifarious problems on the emotions, mental health and well-being of HCWs, although understandably serious, are poorly understood [9]. While some studies have sought to investigate the emotional well-being and health of HCWs in high-income countries [10], there has been a notable scarcity of research dedicated to mid- and low-income nations such as Iran in this regard. This study aimed to address this research gap by exploring various aspects within the context of Iran, including the fear of COVID-19, the responses of healthcare professionals in terms of coping mechanisms during the pandemic, and the positive emotions experienced.

While fear is a natural human emotion with an adaptive function, it can become maladaptive in the face of uncertain situations, heightened intensity, and increased frequency. According to the Lazarus Stress and Coping adaptation model, the experience of fear and stress is defined as “a universally experienced response to extraordinary life circumstances” [11, 12]. Research has suggested that healthcare staff often employ stress and burnout reduction techniques, also known as coping strategies, such as seeking social support and engaging in resilience-promoting interventions to alleviate anxiety, fear, and stress while maintaining their mental health [13, 14]. The coping strategies are important psychological resources can significantly influence the outcomes of coping [11]. Coping self-efficacy has been defined as “the perceived capacity to manage an individual’s personal functioning and adapt to the environmental demands while managing stressful situations” [15, 16].

Numerous studies have underscored that addressing fear, anxiety, and depression often necessitates employing diverse coping strategies, primarily centered on action planning to mitigate associated risks, ultimately resulting in improved emotional well-being [17]. Moreover, an individual’s capacity to stop/steer negative emotions could act as a protective strategy against trauma and stress [18]. Various theorists [13, 19] have highlighted that resilience is inversely correlated with fear, stress, and negative emotions. Individuals tend to exhibit greater resilience in the face of adversity when they possess stronger coping mechanisms [20, 21]. Resilience has been identified as a moderator for adaptive coping strategies, leading to an increase in positive emotions and, consequently, enhancing individual well-being [22].

The stress adaptation framework [11] rooted in transactional theory considers the interplay of personal variables (such as goals, values, and beliefs) and environmental variables (including available resources, demands of stressors, levels of uncertainty, and constraints). These factors influence the mediating processes of stress appraisal and the utilization of coping mechanisms and social support. These mediating processes have an impact on short-term outcomes, encompassing somatic and psychological reactions, as well as long-term effects, including physical and emotional well-being, as well as social functioning. While the theoretical stress adaptation framework has been empirically tested in various contexts, such as dementia [23], autism and morbidity [24], and marital satisfaction [25], it has not, to the best of our knowledge, been used to explore coping strategies of health care professionals, especially in the pandemic setting [26, 27].

With this understanding, the current research examined the following relationships for healthcare professionals in Iran during pandemic times.

- The association of pandemic fear with self-coping strategy.
- The association of pandemic fear with positive emotions.
- The relationship of self-coping strategy with positive emotions.
- The moderating role of resilience between fear of COVID − 19 and self-coping strategy.
- The moderating role of resilience between fear of COVID − 19 and positive emotions.
- The moderating role of resilience between self-coping strategy with positive emotions.
- The mediating role of self-coping strategy in the relationship between fear of COVID-19 and positive emotions.

**Fig. 1:**
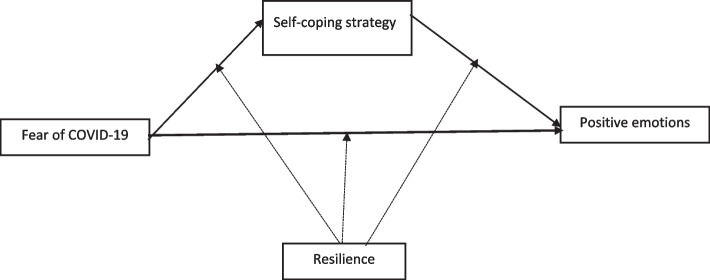
Research model

These objectives are illustrated in the proposed research model (See Fig. 1).

## Methods

### Study design

The study followed a cross-sectional design, involving the collection of data from 1050 healthcare professionals based in Anmol city, situated in northern Iran.

### Settings and sampling

Amol city, situated in the northern region of Iran within Mazandaran province, has hospitals affiliated with Mazandaran University of Medical Sciences. The research population for this study encompassed healthcare professionals employed in these four government hospitals. At the time in which this research was carried out, the total count of healthcare professionals across these four university hospitals was 1155. However, 105 healthcare professionals were excluded from the study due to medical issues affecting data collection.

Upon obtaining ethical approval, the hospital management was contacted and written content was secured from the hospital directors and nursing managers. Subsequently, the researcher visited all departments of the four hospitals and explained the study’s purposeInterviews were conducted with the hospital management at all four hospitals to clarify the study’s objectives and secure consent. A comprehensive list of these healthcare professionals was drawn. Mobile phone numbers of the selected healthcare professionals were recorded verbally, and written consent was obtained through SMS messages.

After receipt of informed consent of participation from the healthcare professionals, they were sent an SMS containing a link to an online survey, which served as the sampling method for data collection.

#### Inclusion/exclusion criteria

To be eligible for participation in the survey, individuals had to meet the following inclusion criteria: (i) the participant must be of Iranian nationality, (ii) the participant must have had a presence in a COVID-19 ward for a minimum of 6 months prior to the study commencement, and (iii) the participant should not have a documented history of mental illness. Participants failing to meet these inclusion criteria were excluded from the study.

### Data collection procedure

The online survey questionnaire was developed using an online “Questionnaire google Doc”.

The sampling method employed in this study was a census approach, i.e., data were collected from all eligible participants. The data collection process involved the use of an online questionnaire, which was distributed to all eligible healthcare professionals via SMS. This method was chosen in light of the COVID-19 pandemic and the imperative to adhere to health protocols. Prior to data collection, a comprehensive explanation of the study’s implementation was provided.

Data collection was carried out during two time frames: the first round coincided with the spread of the Alpha variant of the virus in June 2021, while the second round of data collection was during the spread of the Delta variant phase in September 2021. For the Alpha variant, the questionnaire link was sent once, and for the Delta variant phase, the questionnaire link was sent three times to healthcare professionals. An additional round of questionnaire distribution took place between the fourth and fifth waves and between the Alpha and Delta variants in the northern region of Iran. The Mazandaran University of Medical Sciences commenced vaccination efforts for healthcare professionals in February 2021. Therefore, by the time of data collection during the fourth wave, all healthcare professionals had already received at least one dose of imported vaccines. Participation in the study was contingent upon providing informed consent, and those who did not grant consent were unable to take part. The response rate from participants reached 85%.

### Measures

#### Response to Stressful Experiences Scale (RSES-4)

To assess psychological resilience, the Response to Stressful Experiences Scale (RSES-4) was used [28]. The RSES-4 is a 4-item scale. It was derived from the 322-item Response to Stressful Experiences Scale (RSES-22) [29]. The RSES-4 was based on a five-point Likert scale ranging from 0 = not at all to 4 = exactly like me. The Cronbach’s alpha coefficient was 0.82.

#### Fear of COVID-19 scale (FCV-19 S)

To evaluate the level of fear of COVID-19 “Fear of COVID-19 scale” (FCV-19 S) was used [30]. The scale consisted of seven items (e.g., “I am afraid of losing my life because of coronavirus-19”) and the response was collected based on a five-point Likert scale ranging from 1 = strongly disagree to 5 = strongly agree. The Cronbach’s alpha coefficient reported was 0.92.

#### Positive emotions scale

The positive emotional state was assessed using 5-item scale [31]. This questionnaire contained 5 statements and was tested on a five-point Likert scale (I completely disagree, I disagree, I have no opinion, I agree and I completely agree). A sample item was “I have a very pleasant life”. The value of Cronbach’s alpha coefficient for this was 0.92.

#### Self-coping strategy

The self-coping strategy was assessed using a 26-item scale [32]. A sample item was “Talk positively to yourself”. Anchor points on the scale were 0 (‘cannot do at all’), 5 (‘moderately certain can do’) and 10 (‘certain can do’). The value of Cronbach’s alpha coefficient for this was 0.89.

### Ethical approval and informed consent

The study protocol was approved by the ethics committee of Mazandaran University of Medical Sciences (approval code: IR. MAZUMS.REC.1400.879), and in order to obtain informed consent, an SMS was first sent to all participants via SMS, then after the participants’ approval, a link to the questionnaire was sent to them.

### Data analysis

Structural equation modelling (SEM) methods as implemented in AMOS 24.0 [33] were used to analyze the model. The goodness of fit of the models was assessed based on several indicators, including relative χ^2, root mean square error of approximation (RMSEA), standardized root mean square residual (SRMR), comparative fit index (CFI), and Tucker-Lewis index (TLI). The criteria considered were χ^2^/df < 3, RMSEA ≤ 0.08, SRMR ≤ 0.06, CFI ≥ 0.90, and TLI ≥ 0.90 [34, 35]. Cronbach’s α, variance extracted (AVE), composite reliability (CR), and average loadings (AL) were also analysed to check for reliability and validity of the instruments.

## Results

### Reliability and validity analysis

Table 1 shows the demographic characteristics of the respondents. Table 2 describes the descriptive statistics, average loadings, AVE and composite reliabilities of all variables.

**Table 1:** Demographic characteristics of respondents

Variables	Frequency, Percentage
Socio-demographic information	
Age (Mean ± SD)	33.58 ± 8.02
*Gender*	
Female	797 (75.9%)
Male	253 (24.1%)
*Marital Status*	
Single	296 (28%)
Married	754 (72%)
*Education level*	
Diploma	139 (13.3%)
Bachelor’s degree	759 (72.3%)
Post graduation degree -Medical	116 (11.1%)
Specialist or PhD	35 (3.3%)
*Designation*	
General Practitioner	41 (3.9%)
Specialist	35 (3.3%)
Assistant nurse	107 (10.2%)
Operating room technician	31 (3%)
Anesthesia technician	23 (2.2%)
Laboratory technician	74 (7%)
Radiology technician	15 (1.4%)
Nurse	703 (67%)
Practical nurse	21 (2%)

**Table 2:** Means, standard deviations, and intercorrelations among study variables (*N* = 1050)

Variables	Mean	SD	AL	CR	AVE	1	2	3	4
1. Fear of COVID-19	2.34	0.88	0.77	0.83	0.79	0.88			
2. Self-coping strategy	4.22	0.79	0.79	0.84	0.77	-0.22*	0.87		
3. Resilience	4.11	0.99	0.83	0.79	0.68	-0.23**	0.34***	0.82	
4. Positive emotions	3.99	0.95	0.84	0.83	0.81	-0.19*	0.27**	0.31**	0.90

*SD *Standard Deviation, *AL *Average Loading, *CR* Composite Reliability, *AVE* Average Variance Extracted

*p < 0.05, **p < 0.01, ***p < 0.001

In the initial assessment, internal consistency, convergent validity and discriminant validity of all the variables were analyzed to check the measurement properties of all constructs. Table 2 shows the psychometric properties of all measures considered for the study. The items loadings were above 0.60 and the average variance extracted (AVE) values were above 0.50. This indicated excellent content and convergent validity respectively of all the measures. The composite reliabilities of all the latent variables were above 0.75 and were deemed adequate. The values of Cronbach’s alphas ranged above the critical level of 0.70 [36].

Next, discriminant validity shown in Table 2, represented the correlation matrix for all the constructs. The diagonals show the square root of AVE’s. The square roots of all AVE scores were more than their corresponding inter-correlations and this established discriminant validity. Based on the above values, it can be inferred that the measurement model exhibited an adequate level of reliability and validity.

#### Structural equation modelling results

The results of the SEM analysis demonstrated a good fit to the data, with the following fit indices: χ2/df = 1.71, *p* < 0.01, CFI = 0.95, TLI = 0.95, RMSE = 0.04, and SRMR = 0.05.

The majority of the relationships were found to be both significant and acceptable. You can find the results of the structural equation modeling in Table 3.

**Table 3:** Structural equation modelling results

Relationships	Standardized path coefficient	Result
Fear of COVID-19 ➔ Self-coping strategy	-0.17***	Supported
Self-coping strategy ➔ Positive emotions	0.34**	Supported
Fear of COVID-19 ➔ Positive emotions	-0.27*	Supported
*Mediation (Bootstrap sample size = 2000)*		
Fear of COVID-19➔ Self-coping strategy◊ Positive emotions	-0.19**	Supported
*Moderation*		
Fear of COVID-19 x Resilience ➔ Self-coping strategy	0.07^ns^	Not supported
Self-coping strategy x Resilience ➔ Positive emotions	0.37***	Supported
Fear of COVID-19 x Resilience ➔ Positive emotions	-0.21**	Supported

*N* = 1050, *p < 0.5, **p < 0.01, ***p < 0.001, *ns *Non Significant

The fear of COVID-19 exhibited significant negative associations with both self-coping strategy (β= -0.17, *p* < 0.001) and positive emotions (β = -0.27, *p* < 0.05). On the other hand, the self-coping strategy showed a significant positive relationship with positive emotions (β = 0.34, *p* < 0.01).

We also conducted a partially mediated model for the mediation analysis with a bootstrap sample of 2,000. As presented in Table 3, the self-coping strategy positively mediated the relationship between fear of COVID-19 and positive emotions, with β = 0.09, *p* < 0.001, resulting in a total effect of β= -0.19, *p* < 0.01.

**Fig. 2:**
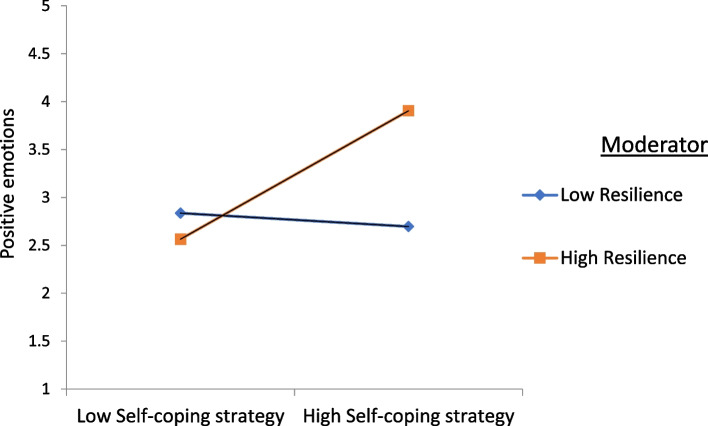
Interaction of resilience on association of positive emotions and self-coping strategy

**Fig. 3:**
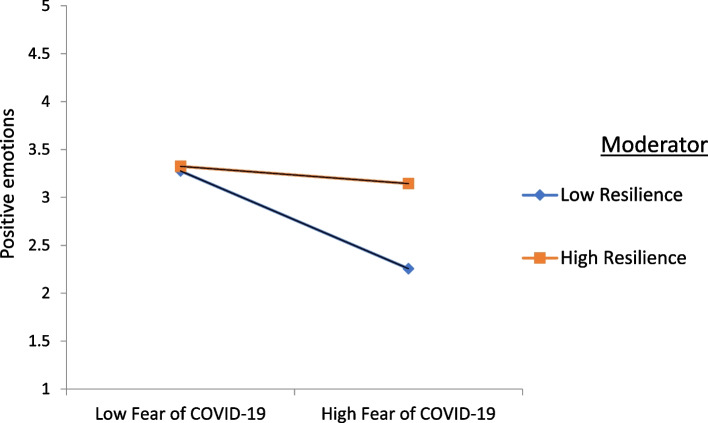
Interaction of resilience on association of positive emotions and fear of COVID-19

The resilience factor did not have a significant moderating effect on the relationship between fear of COVID-19 and self-coping strategies. However, the moderating effect of resilience on the relationship between fear of COVID-19 and positive emotions, and the relationship between self-coping strategies and positive emotions, were found to be significant and positive. Figures 2 and 3 illustrate the moderating effects of resilience.

## Discussion

The current study investigates the interrelationships among pandemic fear, self-coping strategies, resilience, and positive emotions within the context of healthcare professionals in Iran. This study makes several valuable contributions to the existing literature. Firstly, it includes the concept of pandemic fear in positive psychology literature, and offers insights into this connection in the context of the “new normal” scenario [37]. In doing so, it aligns with previous research that has explored resilience during the pandemic [38–40]. Additionally, it extends the body of work that underscores the significance of self-coping strategies among healthcare professionals during a pandemic [41, 42].

The study’s findings reveal negative associations between the fear of COVID-19 and both self-coping strategies and positive emotions. These results corroborate prior research, which has consistently reported negative relationships between pandemic fear and positive outcomes within the general population [43, 44], including among healthcare professionals [45–47]. The study also highlights a robust positive relationship between self-coping strategies and positive emotions, reinforcing findings from similar studies while expanding the scope to include healthcare professionals [48].

The most significant novelty of this work lies in its noteworthy moderation results, particularly the role of resilience in moderating the associations between self-coping strategies, fear of COVID-19, and positive emotions among healthcare workers. While previous studies have explored resilience during the pandemic, this study stands as a pioneering effort, demonstrating the significant moderating influence of resilience, thus potentially advancing the field of positive psychology [49, 50].

Another notable aspect of this study is the incorporation of fear, self-coping strategies, and resilience to understand positive emotions [51, 52]. This addresses an existing gap in knowledge in this area, as previous studies on resilience and positive emotions during the pandemic often overlooked the crucial role of fear. The present study helps fill this void, offering valuable insights into the complex interplay of these factors.

This study can serve as a foundation for future investigations on the positive psychological dimensions of pandemics and other global health crisis situations. Its relevance can be extended to the “new normal,“ characterized by ongoing uncertainty and the persistent fear of recurring events. It highlights the importance of working professionals developing self-coping mechanisms alongside resilience to effectively address and manage pandemic-related fear [53].

This study has several clinical, practical and societal implications as the research focuses on fear of COVID-19 and the coping strategies [54, 55]. In a world continually grappling with a multitude of challenges, including economic uncertainties and the fear of recurring COVID-19 waves, addressing mental health concerns and preventing self-stigmatization due to unprecedented circumstances is a priority [43, 56]. articularly, healthcare professionals face the daunting fear of infection, not only for themselves but also for their loved ones, as they are consistently surrounded by patients who may be severely infectious [57, 58].

In clinical practice, healthcare practitioners can draw insights from this study to reevaluate and expand upon practical applications during the pandemic [18, 59, 60]. Given that clinical practice has been largely restricted to online or telephonic platforms due to pandemic restrictions, practitioners may emphasize advising self-coping strategies and therapies in this context [61, 62]. These strategies could potentially include techniques like self-talk, physical exercises, yoga, proper sleep, and dietary recommendations [61, 63]. A collective approach can be considered wherein individuals support each other with collective coping techniques to mitigate the risk of mental health disorders [64, 65].

Training or mindfulness-based coping strategies like breathing exercises, and RAIN meditation can be used to promote and enhance the mental health of healthcare professionals by bolsering positive emotions [66]. Furthermore, governmental authorities can offer support to healthcare professionals by providing psychological assistance to those at a higher risk of developing mental health issues [67]. The overarching aim is to cultivate greater resilience, as the environment and circumstances consistently undergo change. In this context, nurturing positive emotions can significantly contribute to improved mental health, especially among healthcare professionals during such challenging periods [9].

There are a few limitations in this study. Firstly, the data was collected via self-reported measures and therefore, carried the risk of common method bias. Secondly, the research used cross-sectional data and and therefore, conducting a longitudinal study could offer more comprehensive insights. Additionally, future research may benefit from conducting cross-cultural comparisons. Further, exploring additional variables like happiness, optimism, and various forms of support among others could enhance the depth of understanding in future studies. Investigating the moderating roles of factors such as gender and age could also be a valuable avenue for research to gain insights into their potential impact.

## Conclusion

This study which involved responses from 1050 healthcare professionals in Iran, showed that pandemic fear has a detrimental effect on self-coping strategies and positive emotions in crisis situations. Conversely, self-coping strategies exhibit a positive correlation with positive emotions. Significantly, the research also highlighted that resilience amplifies the favorable connection between self-coping strategies and positive emotions.

The study’s findings can guide future research in this field and have practical applications, particularly in the context of the “new normal” characterized by ongoing uncertainty and crises. These insights can help in promoting greater positivity and well-being among healthcare professionals.

## Data Availability

The datasets used and/or analysed during the current study available from the corresponding author on reasonable request.
